# Maternal and Perinatal Outcomes of Hospitalized COVID-19 Positive Pregnant Women

**DOI:** 10.7759/cureus.21817

**Published:** 2022-02-01

**Authors:** Vandana Gupta, Yogesh Yadav, Reena Sharma, Manish Mishra, Diksha Ambedkar, Vani Gupta

**Affiliations:** 1 Obstetrics and Gynaecology, Rajarshi Dashrath Autonomous State Medical College, Ayodhya, IND; 2 Pathology, Rajarshi Dashrath Autonomous State Medical College, Ayodhya, IND; 3 Biochemistry, Rajarshi Dashrath Autonomous State Medical College, Ayodhya, IND; 4 Physiology, King George's Medical University, Lucknow, IND

**Keywords:** perinatal outcome, maternal outcome, new-born, pregnant women, covid-19

## Abstract

Introduction

The consequences of severe acute respiratory syndrome coronavirus 2 (SARS-CoV-2) /Coronavirus disease 2019 (COVID-19) on mothers and neonates are uncertain due to the lack of robust evidence from various available studies. Furthermore, conflicting data exist regarding the vertical transmission of coronavirus. Therefore, a hospital-based study was conducted to evaluate the effect of COVID-19 on maternal and perinatal outcomes of COVID-19 infected pregnant women.

Methodology

A hospital-based retrospective observational study was conducted between July-December 2020 in Rajarshi Dashrath Autonomous State Medical College, Ayodhya, Uttar Pradesh, a designated level-2 COVID-19 Hospital. A total of 37 confirmed COVID-19 positive pregnant women (mean age 27.5 ± 05 years) of more than 28 weeks of gestation were included in this study to evaluate the effect of COVID-19 on maternal and perinatal outcomes. Maternal symptoms related to COVID-19, comorbidities, intensive care unit (ICU) admissions, intrauterine growth retardation (IUGR), leaking per vagina, mode of delivery, preterm deliveries, and maternal deaths were recorded. Birth weight of newborns, neonatal intensive care unit (NICU) admissions, neonatal illness, neonatal deaths, and COVID-19 testing reports were recorded.

Result

Out of 37 COVID-19 positive pregnant women, 27 (72.9%) women were asymptomatic, nine (24.4%) women were having mild disease, and one (2.7%) developed severe disease requiring ICU admission. No maternal deaths were observed. Twenty-six (70.3%) women were delivered by caesarean section, 11 (29.7%) women by normal vaginal delivery, four (10.8%) were of leaking per vagina. Among newborns, five (13.5%) were preterm, one (2.7%) newborn require NICU admission, two (5.4%) were tested COVID-19 positive on the 5th day of life but were asymptomatic, and four (10.8%) newborns developed a fever but were COVID-19 negative. One case (2.7%) was of stillbirth. No neonatal deaths were observed.

Conclusion

The present study did not reveal any direct evidence for vertical transmission of SARS-CoV-2 virus through the placenta and during vaginal delivery, but the possibility of mother-to-child infection cannot be completely ignored. SARS-CoV-2 infection during late pregnancy may have a maternal and neonatal impact. COVID-19 infections in late pregnancy might lead to an increased incidence of caesarean deliveries as observed in the present study. This study reveals that most of the COVID-19 positive pregnant women remained asymptomatic or had mild infections. Hence, efforts to limit exposure to COVID-19 of pregnant women should be strengthened for saving mother and child.

## Introduction

COVID-19 was declared a pandemic by the WHO on 11 March 2020 [1]. It is an immunogenic, thrombogenic, inflammatory, contagious viral disease caused by severe acute respiratory syndrome coronavirus-2 (SARS-CoV-2). Immunological changes during pregnancy may induce a state of increased susceptibility to certain intracellular pathogens, including viruses, bacteria, and parasites [2]. Pregnant women might be more susceptible to SARS-CoV-2 infection [3]. Some studies observed that SARS-CoV-2 infection although rare in pregnant women, was higher than similarly aged adults in Washington State [4], Compared to other viral diseases the immunogenic, thrombogenic, and inflammatory nature of SARS-CoV-2 are threats and may lead to adverse deleterious consequences in the pregnant women herself or to birth adverse outcomes such as miscarriage, intrauterine death, fetal growth restriction. High case fatality rates have been associated with SARS and MERS [5]. Evidence from other coronavirus infections, such as SARS-CoV or MERS-CoV, suggests that infected pregnant women might be more susceptible to adverse outcomes, including intubation, intensive care unit (ICU) admission, renal failure, and death [6]. SARS-CoV-2 infection may be detrimental in pregnancy [7]. However, the clinical course of COVID-19 pneumonia in pregnant women has been reported to be similar to that in non-pregnant women. The association between COVID-19 positive pregnant women and the risk of adverse maternal & perinatal outcomes is not very clear. Vertical transmission like other viral diseases including HIV, ZIKA, CMV, etc, during pregnancy, at the time of childbirth, or during breastfeeding may be a possibility. Conflicting data exist regarding the vertical transmission of the virus [8-10]. Till now, very few studies are available regarding this in Uttar Pradesh. This hospital-based study was conducted to evaluate the effect of COVID-19 on maternal and perinatal outcomes of COVID-19 infected pregnant women.

## Materials and methods

A hospital-based retrospective observational study was conducted between July 2020 - December 2020 in Rajarshi Dashrath Autonomous State Medical College, Ayodhya, Uttar Pradesh, designated as a level-2 COVID-19 hospital. A total of 37 COVID-19 positive pregnant women (mean age 27.5 ± 5 years) of more than 28 weeks of gestation were included in this study to evaluate the effect of COVID-19 on maternal and perinatal outcomes. COVID-19 negative pregnant women and COVID-19 positive pregnant women who were less than 28 weeks of gestation or not delivered in this hospital were excluded. Maternal symptoms related to COVID-19, comorbidities, intensive care unit (ICU) admission, leaking per vagina, intrauterine growth retardation (IUGR), mode of delivery, preterm deliveries, birth weight of newborns, neonatal congenital anomalies, neonatal intensive care unit (NICU) admission, maternal and neonatal deaths were recorded. All the neonates were kept under observation for seven days for the development of any kind of illness. COVID-19 test (SARS-CoV-2 RT-PCR test) was done on nasopharyngeal swabs from all neonates on the first day of life (at 24 hrs after birth), and thereafter on the fifth and seventh day of life. All newborns were allowed for breastfeeding. The newborn and his/her mother were kept in the same room on separate beds maintaining proper social distancing along with one apparently healthy asymptomatic (having RT-PCR negative report) caregiver, for taking care of the newborn. Neonates were handed over to mothers only during breastfeeding. Mothers were instructed to take general COVID-19 preventive measures including wearing a surgical face mask during breastfeeding and practicing hand hygiene (hand wash/sanitization) before each breastfeeding. The caregiver was also instructed to wear a face mask and maintain hand hygiene. The caregiver was monitored for COVID-19 infection during the hospital stay and was also subjected to COVID-19 testing at the time of discharge or on becoming symptomatic. Management of COVID-19 infected pregnant women, caregivers if they became infected, and newborns were done as per standard guidelines. 

## Results

Among 37 COVID-19-positive pregnant women, 18 (48.6%) were primiparous and 19 (51.4%) were multiparous (Table 1).

**Table 1:** Distribution of cases according to parity

S.No.	Parity	Number	Percentage
1.	Primiparous	18	48.6%
2.	Multiparous	19	51.4%

Out of 37 COVID-19 positive pregnant women, one woman (2.7%) had gestational diabetes controlled by diet, four (10.8%) were anemic, two (5.4 %) had hypothyroidism, and one (2.7%) was hypertensive and controlled on antihypertensive medications (Table 2).

**Table 2:** Distribution of cases according to comorbidities

S.NO	Comorbidities	Number	Percentage
1.	Gestational diabetes	01	2.7%
2.	Anaemia	04	10.8%
3.	Hypothyroidism	02	5.4%
4.	Hypertension	01	2.7%

Out of 37 COVID-19-positive pregnant women, 26 women (70.3%) were delivered by lower segment caesarean section (LSCS) and 11 (29.7%) were delivered by normal vaginal delivery. Five (13.5%) were of preterm (< 37 weeks of gestation) deliveries, 32 women (86.5%) were delivered at term gestation (≥ 37-42 weeks of gestation) including 12 (32.4%) women of post-dated deliveries ( >40 weeks of gestational age). Four (10.8%) women had leaking per vagina. No case of intrauterine growth retardation was reported. Out of 37 COVID-19-positive pregnant women, 27 women (72.9%) were asymptomatic, five women (13.5%) had fever and myalgia including two (5.4%) women who had headaches, and four (10.8%) women had complaints of cough. One (2.7%) woman who was hypertensive developed breathlessness with the decrease in SpO_2_ to 68% on the second day of the caesarean section, required ICU admission, recovered after management, and was shifted back to the room after two days. No maternal deaths were reported (Table 3).

**Table 3:** Maternal outcomes

S.No.	Maternal Outcomes			Number	percentage
1.	Gestational age at the time of delivery	Term gestation (37-42 weeks)		32	86.5%
		Preterm gestation (<37 weeks )		05	13.5%
2.	Mode of delivery	Delivery by LSCS		26	70.3%
		Normal vaginal delivery		11	29.7%
3.	Leaking per vagina			04	10.8%
4.	Meconium stained liquor			01	2.7%
5.	Intrauterine growth retardation			00	0.0%
6.	COVID -19 status		Positive	37	100%
			Negative	00	0.0%
7.	Maternal symptoms related to COVID – 19		Asymptomatic	27	72.9%
			Fever & Myalgia	05	13.5%
			Cough	04	10.8%
			Headache	02	5.4%
			Severely ill (ICU admission)	01	2.7%
8.	Maternal deaths			00	0.0%

Among neonates birth weights of five (13.5%) newborns were less than 2.5 kg, six (16.2%) newborns were of 2.5-2.8 kg, nine (24.3%) newborns were of 2.8-3.0 kg, 13 (35.1%) were of 3.1-3.5 kg, and four were of 3.6-4.0 Kg.

All neonates (100%) of the 37 COVID- 9 positive women were tested for COVID-19 by SARS-CoV-2 RT-PCR test (gold standard test for COVID-19) on the first day of life, i.e at 24 hrs after birth, and on the fifth, and seventh day of life by nasopharyngeal swabs. All neonates (100%) tested COVID-19 negative (including one stillbirth) at 24 of birth, but two (5.4%) became COVID-19-positive on the fifth day of their life, one of them became COVID-19 negative on the seventh day of life, while the other one remained COVID-19 positive even on the seventh day of life. However, on follow up both neonates remained asymptomatic and not required any additional care or NICU admission.

COVID-19 infection to these neonates might be droplet infection or through the vagina in one neonate during vaginal delivery. Four neonates, although COVID-19 negative, developed fever which subsided with treatment. One neonate required NICU admission because of meconium aspiration. Five (13.5%) newborns were of preterm birth. There was one (2.7%) case of stillbirth attributed to obstetrical reasons. No obvious neonatal congenital abnormalities were reported (Table 4). 

**Table 4:** Neonatal outcomes

S.NO.	Neonatal outcomes			Number	Percentage
1.	Birth weight of new-born ( in kilogram )		<2.5 kg	05	13.5%
			2.5 - 2.8 kg	06	16.2%
			2.8 - 3.0 kg	09	24.3%
			3.1 - 3.5 kg	13	35.1%
			3.6 - 4.0 kg	04	10.8%
2.	NICU admission			01	2.7%
3.	Fever			04	10.8%
4.	Preterm birth			05	13.5%
5.	Stillbirth			01	2.7%
6.	Congenital abnormalities			00	0.0%
7.	COVID -19 status By nasopharyngeal RT-PCR	No neonate positive at 24 hrs of birth including one stillbirth		00	0.0%
		Two neonates became positive on the fifth day of life		02	5.6% (calculated after excluding one stillbirth)
		One neonate positive on the seventh day of life		01	2.8% (calculated after excluding one stillbirth)

In the present study, only one caregiver, who roomed in with severely ill COVID 19 positive women, became COVID 19 positive when tested on the day of discharge, but remained asymptomatic.

## Discussion

In this study, it was observed that 72.9 % of COVID-19 positive pregnant women were asymptomatic. Fever and myalgia reported in 13.5% of cases were the most common presenting symptoms, followed by cough in 10.8% of cases. Other studies also reported fever as the most common symptom followed by a cough [5,8,11]. The present study showed that 2.7% of cases with underlying comorbidity of hypertension developed a more severe course and progression of COVID-19 disease. In this case, the mother developed breathlessness with a decrease in SpO_2_ to 68 % on the second postoperative day of caesarean delivery and was shifted to the ICU. Similar findings were also reported by Mazur-Bialy et al. [9]. Increased incidence of caesarean delivery (70.3%) was observed in COVID-19 positive pregnant women in the present study. High caesarean rates in COVID-19 infected pregnant women were also observed in some other studies [11,12]. 

A total of 13.5% cases of preterm birth were reported in this study with no case of early preterm birth (less than 32 weeks gestational age) was observed. All cases were of spontaneous preterm delivery. In a systematic review, Smith et al. reported 63.8% preterm birth and attributed that to viremia [11]. The possible explanation for our findings of preterm cases in the present study may be the high prevalence of asymptomatic or mild cases of COVID-19 pregnant women where viremia was insufficient to trigger parturition cascade. The findings of the present study corroborate with the other studies [13,14].

In this study, 10.8% of women presented with leaking per vagina. Similar findings were also reported by Kalpana et al. [12]. 

In the present study, low birth weight (birth weight < 2.5 kg ) was reported in 13.5% of newborns. No newborns had a birth weight below 2.3 kg. Birth weight in 86.4% of newborns was ≥ 2.5 kg. Kalpana et al. [12] also reported that 20.7% of newborns of COVID-19 positive mothers had birth weight between 1.5 to 2.4 kg, and 72.4% had birth weight ≥ 2.5 kg. 

In the present study, 100% of neonates were roomed in with mothers and allowed for breastfeeding. No neonates were reported COVID-19 positive on the first day of their life i.e at 24 hr of birth, which rules out vertical transmission in utero from COVID-19 positive mothers during the third trimester. None of the neonates developed respiratory distress. Observations of the present study are congruent with the study by Wang et al. [5] which also reported no vertical transmission. However, another study by Dong et al. [15] shows possibilities of vertical transmission. A study revealed that the requisite machinery is expressed in the placenta right from the first trimester. The mRNAs coding for the SARS-CoV‑2 binding receptor (ACE2) and the proteases needed for viral entry and proteins needed for viral multiplication were found to be expressed by certain subtypes of placental cells, which are involved in key placental functions [16]. One systematic review of 93 pregnant women with COVID-19, also showed that within this cohort, nearly 12 % had placental infection with SARS-CoV‑2, suggesting a possibility of vertical transmission of the virus [17]. In the present study, we observed COVID-19 infection detected positive on the fifth day of life in only two neonates (5.4%). One of them delivered through normal vaginal delivery of a woman having cough, while another one through caesarean delivery of a woman who developed breathlessness with SpO2 68 % on the second postoperative day. Additionally, the caregiver for the baby in the latter case became COVID-19 positive on the day of discharge but remained asymptomatic. The caregiver, in this case, may have been infected by the mother or by her baby because of some breach in the preventive measures. As we have not confirmed the cervical, vaginal, and anal COVID-19 virus presence through cervical, vaginal, and anorectal swabs sampling, the possibility of virus presence is there as reported in other studies that COVID-19 viral load has been detected in the vagina, cervical [18] and in anorectal [19] swab sampling of COVID-19 pregnant women. In this study, four (10.8%) newborns remained COVID-19 negative till the seventh day of their life but developed a fever on the second day of life onward; this fever subsided by treatment. One of the possibilities behind this fever might be mild to moderate COVID-19 infection although SARS -CoV-2 RT-PCR test was negative for these four newborns as the sensitivity of this test is 63% for nasal swabs [20]. Additionally, the pregnant women studied were not suffering from a severe grade of SARS-CoV-2 infection. Neonates repeatedly testing negative on nasopharyngeal swabs is difficult to explain, as these tests are not always positive with infection [10]. COVID-19 test was not done for amniotic fluid, placental tissue, cord blood, and breast milk. Neonates may be infected from the mother via droplet infection (horizontal transmission). Although the present study did not reveal any direct evidence for vertical transmission of the SARS-CoV-2 virus through the placenta and during vaginal delivery, the possibility of mother-to-child infection cannot be completely ignored. 

The limitations of this study were the absence of data on the amniotic fluid, cord blood, vaginal secretion, and breast milk samples, as all resources were stretched in a pandemic. Moreover, no data about the first or second trimester of pregnancy was reported, since COVID-19 infection in different trimesters might be associated with different outcomes.

## Conclusions

In conclusion, the present study did not reveal any direct evidence for vertical transmission of the SARS-CoV-2 virus through the placenta and during vaginal delivery, but the possibility of mother-to-child infection cannot be completely ignored. COVID-19 infections in late pregnancy might lead to an increased incidence of caesarean deliveries as observed in the present study. This study reveals that most of the COVID-19 positive pregnant women remained asymptomatic or had a mild infection. COVID-19 infection during late pregnancy may have a maternal and neonatal impact. Conclusively, efforts to limit exposure of pregnant women should be strengthened for saving mother and child. As this is a single-center study, multicentric studies on a larger sample size including all trimesters of pregnancy are required for a complete evaluation of COVID-19 impact on pregnancy and its outcomes.
